# The price of private dental services: results from a national representative survey of Ireland

**DOI:** 10.1007/s11845-022-03041-7

**Published:** 2022-06-29

**Authors:** Samantha Smith, Jing Jing Jiang, Charles Normand, Ciaran O’Neill

**Affiliations:** 1 Centre for Health Policy & Management, School of Medicine, Trinity College Dublin, 3-4 Foster Place, Dublin 2, Ireland; 2 Cicely Saunders Institute, Bessemer Road, London, SE5 9PJ UK; 3 Centre for Public Health, Queen’s University Belfast, Royal Victoria Hospital, Belfast, BT12 6BA Northern Ireland

**Keywords:** Dentists, Dental examination, Dental practices, Inequalities, Ireland, Oral health, Price, Survey

## Abstract

**Introduction:**

Dental services in Ireland are delivered in a mixed public–private system but the majority of dental care is paid for out-of-pocket by individuals. Ireland is not unusual in the global context where public subsidisation for oral healthcare is limited in many countries. This is despite the fact that oral health plays an important role in well-being and despite international evidence on the negative impact of user fees on utilisation of beneficial healthcare. However, there has been little up-to-date assessment of the prices faced by individuals for a range of non-acute care services in Ireland, including dental care. This paper presents an up-to-date assessment of private dental prices in Ireland for a range of preventive, primary, and complex services based on a nationally representative survey.

**Methods:**

The total sample size for the desk-based survey was 103, accounting for 6% of private dentists in Ireland, weighted to reflect the geographic distribution of dentists. Dentists were selected at random from the publicly available list of dentists participating in the Dental Treatment Benefit Scheme. The adult price of 10 different services covering core preventive, primary, and complex procedures were identified from public websites for the selected dental practices.

**Results:**

Results showed that in addition to there being an uneven supply of dentists across the country, dental prices also vary with some notable variations by region and type of service. In particular, dental practices located in border counties, and those in rural areas typically show lower mean prices relative to non-border counties and urban areas. These factors need to be considered when planning how to reduce inequalities in access to oral health services in Ireland.

## Introduction

Oral health is widely recognised to play an important role in well-being but yet public subsidisation for oral healthcare remains limited in many countries, including Ireland, with high proportions of dental care paid for out-of-pocket by individuals [1]. This is despite international evidence on the negative impact of user fees on the utilisation of beneficial healthcare [2].

In Ireland, dental services are delivered in a mixed public–private system but the majority of dental care is delivered by private sector general dental practitioners and paid for out-of-pocket [3]. High user fees have been observed to impact on access to important primary healthcare services in Ireland [4, 5] and there is a concern that high prices are evident for dental care also, with implications for access and evidence of dental tourism with some Irish patients travelling abroad to pay for cheaper complex dental care elsewhere [6].

While the new national oral health policy, “Smile Agus Sláinte” [7], aims to reduce oral health inequalities across the population, the main focus of the policy is on improving access for children, adult medical cardholders, and vulnerable patients. The rest of the population has limited access to publicly subsidised preventive dental services and pay out-of-pocket for many dental services. Therefore, it is important to examine the prices charged for privately provided dental care and this is the focus of this paper. We present an up-to-date assessment of the private dental prices in Ireland for a range of preventive, primary, and complex services based on a nationally representative survey.

The following sections outline the background structure of dental services in Ireland, the methods used to undertake a desk-based survey of dental price lists in a sample of 103 private dental practices, the survey results, and discussion.

### Structure of dental care in Ireland

The provision of dental care in Ireland was guided until recently by the 1994 Dental Health Action Plan and this was replaced by Smile Agus Sláinte in 2019 [7]. The new policy aims to reorient the provision of oral healthcare services with greater emphasis on prevention [8]. The key goals of the new policy are to put in place the supports necessary to ensure that everyone can achieve their best oral health and to reduce oral health inequalities across the population [7].

**Fig. 1:**
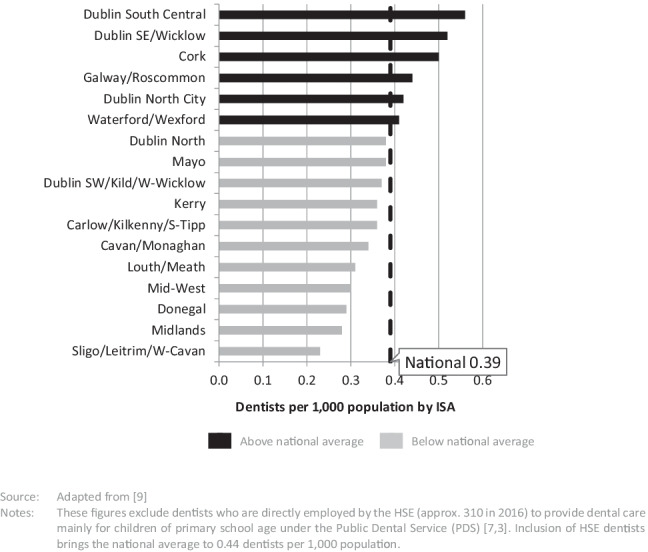
Estimated number of private dentists per 1000 population by ISA, Ireland, 2016. Source: Adapted from [9]. Notes: These figures exclude dentists who are directly employed by the HSE (approx. 310 in 2016) to provide dental care mainly for children of primary school age under the Public Dental Service (PDS) [3, 7]. Inclusion of HSE dentists brings the national average to 0.44 dentists per 1000 population

The majority of dentists work as private practitioners and there are approximately 1785 private dentists currently practising in Ireland (2016 data), most of whom are dental surgeons (95%) with a small number of orthodontists [7, 9]. This is equivalent to 0.39 private dentists per 1000 population, but these are not distributed evenly across the country [7]. Figure 1 shows the estimated distribution of private dentists per 1000 population by Integrated Service Area (ISA).^1^ The number of private dentists varies from 0.23 per 1000 population in Sligo/Leitrim/West Cavan ISA to 0.56 per 1000 population in Dublin South Central ISA.

Varying levels of public subsidisation for dental care are available and many private dentists hold an agreement with the government to provide specified dental treatments to different groups in the population.

The Dental Treatment Services Scheme (DTSS) finances some dental healthcare services for adult medical cardholders and private dentists are paid on a fee-for-service basis for treatments provided under this scheme [11, 12].^2^ The range of treatments covered by this scheme is limited (one oral examination, two fillings, and (any) extractions, per calendar year) although more complex treatments (e.g. dentures, advanced periodontal (gum) treatments) are available where prior approval is provided by the HSE. The fee schedule has remained unchanged since 2013 [7, 12, 14] following cuts adopted in the wake of the financial crisis [3]. For example, fees for non-complex treatments include €33 for oral examination, €31 for prophylaxis, €50–52 for fillings, and €39.50 for routine extractions [14]. Recent analysis shows that the majority of claims in this scheme are for oral examinations, fillings, extractions, and other non-complex treatments [8].

The Dental Treatment Benefit Scheme (DTBS) subsidises a small range of dental treatments for adults without a medical card with eligibility linked to their Pay Related Social Insurance (PRSI) contributions [2]. The DTBS is administered by the Department of Employment Affairs and Social Protection (DEASP) [3]. For individuals with the requisite number of PRSI contributions, the DEASP pays in full for an oral examination once per year and subsidises (to the value of €42) the cost of a scale and polish or the cost of a periodontal treatment (if clinically necessary). If the cost of either of these services is greater than €42, the individual pays the balance. The balance is capped at €15 for a scale and polish, but there is no cap for the balance charged for periodontal treatment.^3^ The majority of claims in this scheme are for oral examinations (50%) and scale and polish (44%) [8].

A small number of dentists (approx. 310 in 2016) are directly employed by the HSE to provide dental care mainly for children of primary school age under the Public Dental Service (PDS) [3, 7]. The service operates targeted clinical assessments of children in 2nd, 4th, and 6th class (approximately ages 7 to 13) and this is reflected in the age profile of attended appointments [8].

Coverage for privately financed dental care is available in some private health insurance packages, and some non-routine procedures are eligible for 20% tax relief (e.g. crowns, root canal treatment, periodontal treatment).^4^ However, recent analysis of national expenditure data indicates that private out-of-pocket payments account for 66% of total (public and private) dental expenditure in Ireland [8].

## Methods

### Survey methods

#### Sample size

The sample was selected to be representative of the number of private dentists (i.e. excluding those directly employed by the HSE) per 100,000 population in each county in Ireland. The total number of private dentists per 100,000 population at ISA level in 2016 [9] was redistributed to county level based on population shares of counties within ISAs.^5^ This involved a staged process whereby ISAs were first disaggregated to Local Health Office (LHO)^6^ level, and from there to county level. For example, Midlands ISA comprised Longford/Westmeath LHO and Laois/Offaly LHO, which in turn comprised the four counties of Longford, Westmeath, Laois, and Offaly. Dublin and Tipperary were further disaggregated into North and South based on population shares. These methods follow those used in a recent study of the county-level distribution of a range of non-acute healthcare services in Ireland [15].

A sample size of 104 was selected to account for 6% of private dentists in the State, weighted to reflect the geographic distribution of dentists. Table 1 presents the breakdown of the sample by county. Similar sample sizes have been used in previous national price surveys of dental and GP fees in Ireland (e.g. samples of 128 dentists and 123 GPs surveyed by the National Consumer Agency in 2010 [16]).^7^

**Table 1:** Estimated supply of private dentists per 100,000 population by county: total number and survey sample, Ireland, 2016

County	Total number of private dentists per 100,000 population	Sample of private dentists per 100,000 population (20% of total dentists per 100,000)
Cork	48	10
Dublin North	39	8
Mayo	38	8
Galway	35	7
Kerry	33	7
Dublin South	31	6
Donegal	29	6
Wexford	23	5
Meath	19	4
Cavan	18	4
Waterford	18	4
Limerick	15	3
Monaghan	15	3
Sligo	15	3
Kilkenny	14	3
Tipperary South	13	3
Louth	12	2
Clare	9	2
Kildare	9	2
Roscommon	9	2
Carlow	8	2
Laois	8	2
Leitrim	8	2
Westmeath	8	2
Offaly	7	1
Tipperary North	6	1
Wicklow	6	1
Longford	4	1
Total sample		104

Sources: Supply of dentists by ISA in 2016 by Haase and James [9]

Disaggregation from LHO to county level and population by county [10, 18]

Dublin North and South are defined in terms of areas north and south of the River Liffey

The population shares used to disaggregate the supply of dentists from ISA to LHO and from LHO to county were based on 2011 census data to be consistent with the examination of the geographic distribution of other non-acute healthcare services over a similar time period [10]

#### Sample selection

The methods for calculating the sample size, and the distribution of the sample by county outlined above were based on the most comprehensive list of dental practitioners available to date for Ireland. This list consolidated data from a number of different sources but has not been updated and is not publicly available. A more recently updated list is available on the names and dental practices of dentists participating in the DTBS. This list is published online by the DOH and was last updated in 2019 and was used for the sample selection. For each county, the requisite number of dentists (as outlined in Table 1) was selected at random from the DOH list.

For each dentist selected, the following steps were taken:

1. Identify the dental practice for the selected dentist
2. Identify the website for the dental practice, where available
3. Record the price data outlined for selected services (outlined below)
4. Where a website was not available for that dental practice, another dentist was identified from the list and steps 2–3 were repeated

The price of 10 different services were included in the survey. These services were selected to be in line with previous dental price surveys but also to capture examples of services of differing levels of complexity from preventative, to primary and more complex procedures as described in the recent national oral health policy [7]. The services are categorised as follows:

- Preventive: oral examination, prophylaxis/cleaning (with dentist or hygienist), X-rays, fissure sealants
- Primary: periodontal care, composite (white) fillings (restoration),^8^ routine extractions
- Complex: surgical extractions, endodontics (root canal treatment)
- Other: teeth whitening kits

Where a price list specified different prices for adults and children/students, the prices for adults were selected. Where a price list indicated a price range for a specific service (e.g. €80–€100 for a routine extraction), the mid-point of the price range for that service was reported in the analysis (i.e. mid-point of €80–€100: €90). For some services, the duration of visit was specified and in the majority of these cases, the duration referred to a 30-min session but where this varied (e.g. 45–min session), the price was adjusted to give the price per 30-min session to facilitate comparison.

#### Methods of analysis

For each service, core descriptive statistics are reported including the mean, standard deviation, range, and median. To facilitate comparison of prices across counties, bar charts and box plots are presented to illustrate skewness and outliers.

Variations across regions were further examined in terms of urban–rural patterns, proximity to the border, and market concentration:

- For urban–rural patterns, dental addresses were assigned one of six categories ranging from city to highly rural/remote area based on an urban/rural classification system developed by the Central Statistics Office that takes into account population density as well as patterns of employment location. For example, a satellite urban town is defined as having a population between 1500 and 49,999 where 20% or more of residents work in cities.^9^ For the purposes of this analysis, the six categories were merged into two groups to compare prices between urban-based practices and rural-based practices.

- For proximity to the border, price comparisons were made between the dental practices located at the border (counties Cavan, Donegal, Leitrim, Louth, Monaghan, and Sligo) and the rest of the country.

- For market concentration, the number of dentists per head of population (i.e. dentist density) in each county was used as an indicator of market concentration. Low and high market concentrations were defined in relation to the national average number of private dentists per 1000 population (i.e. below and above 0.39 dentists per 1000 population). Prices were compared across counties with low and high market concentrations.

All analyses were performed in STATA/SE V. 17.0.

## Results

### Sample selection

The survey was undertaken over the period August–October 2020. The total sample reached was 103 out of the target of 104. In cases where a dentist was selected but no website was available, alternative dentists were selected within the same county. Only in one county, Leitrim, could an alternative dentist not be found and the sample quota was not reached.

### Price data

#### Preventive services

Table 2 presents the mean prices for the preventive services together with summary descriptive statistics. The mean price for an oral examination is €41 (std. €13); cleaning with the dentist is €63 (std. €12), and the estimated mean price for the combined service of examination plus cleaning (with the dentist) is €93 (std. €16).

**Table 2:** Adult prices of preventive services from survey of 103 dental practices, Ireland, 2020

Preventive service	Sample size (*n*)	Price €			
		Min	Max	Median	Mean (std)
Oral examination	103	0^b^	90	40	41 (12.8)
Prophylaxis/cleaning	79	30	95	60	63 (11.5)
Examination and prophylaxis/cleaning^a^	86	60	140	93	93 (15.8)
Cleaning with hygienist (30 min)^c^	50	50	115	70	69 (11.1)
Fissure sealants (per tooth)	65	12.5	50	30	31 (7.6)
Small X-ray	85	0	50	15	18 (9.1)
Large X-ray	66	30	95	50	52 (12.4)

Source: Author’s own calculations based on desk-based survey of dental practice websites, see “Methods” for full description

^a^A combined examination and cleaning price was listed in 45% of the sample, a combined price was calculated for the remainder of the sample (i.e. €exam + €cleaning). The sample size for the combined examination and cleaning price is larger than the sample size for cleaning alone because for some price lists, the price of the combined service was listed while a separate price for cleaning was not

^b^Zero values refer to services that are provided free of charge

^c^Where possible, prices were adjusted pro-rata to reflect a 30-min session but in many cases the duration of the visits were not specified

Where a price list indicated a price range for a specific service the mid-point of the price range for that service was reported in the survey

When outliers are excluded the mean prices for preventive services fall by between 0.2 and 2.4% with the exception of the price for a small X-ray where exclusion of outliers reduces the mean price by €1 or 7.5%

Cleaning with the hygienist is more expensive than with the dentist, with an average price of €69 per half hour session. The mean price for fissure sealants is €31 (std. €8) per tooth. The mean prices for X-rays range from €18 (std. €9) for small to €52 (std. €12) for large X-rays.

In all cases, the mean price is close to the median indicating that the level of skewness in the data is not large.

**Fig. 2:**
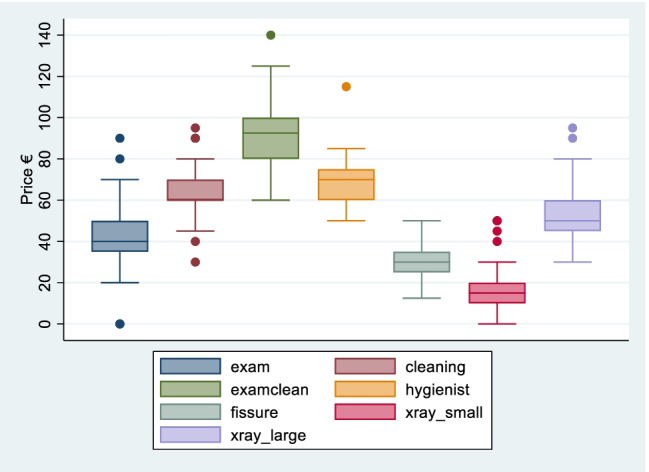
Boxplot of prices for preventive services from survey of 103 dental practices, Ireland, 2020. Source: Author’s own calculations based on desk-based survey of dental practice websites, see “Methods” for full description

Figure 2 presents the boxplots for each of these services to help illustrate the underlying distributions for these prices and dispersion around the median prices. For the more expensive services in this category (combined exam and cleaning; cleaning with hygienist; large X-rays), prices are somewhat more dispersed than for the less expensive services. There are a small number of outlying values for each of the services (with the exception of fissure sealants) but exclusion of outlying values does not have a large impact on the mean price (between 0.2 and 2.4% with the exception of small X-rays where exclusion of outliers reduces the price by 7.5% from €18 to €17) and there are no distinctive regional patterns to where the outliers are located.

#### Primary services

Table 3 presents the mean prices for the primary services. The mean price of a small composite filling is €91 (std. €17) and €148 (std. €44) for a large composite filling. The mean price for a routine extraction is €100 (std. €22) and the mean price for a session of periodontal (gum) care is €104 (std. €36).

**Table 3:** Adult prices of primary services from survey of 103 dental practices, Ireland, 2020

Primary service	Sample size (*n*)	Price €			
		Min	Max	Median	Mean (std)
Composite fillings (restoration):					
Small	97	35	140	90	91 (16.7)
Large	86	85	375	145	148 (44.2)
Extraction (routine)	100	45	185	95	100 (22.1)
Periodontal (gum) care^a^	64	20	200	98	104 (36.3)

Source: Author’s own calculations based on desk-based survey of dental practice websites, see “Methods” for full description

^a^Periodontal care prices were quoted in different ways: per quadrant, per session, by time (e.g. 30 min, 45 min). Where possible, prices by time were adjusted pro-rata to reflect a 30-min session and compared with prices per quadrant and per session

Where a price list indicated a price range for a specific service the mid-point of the price range for that service was reported in the survey

**Fig. 3:**
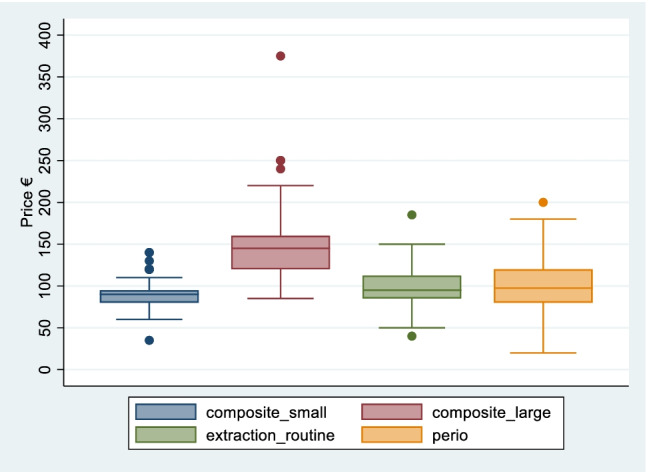
Boxplot of prices for primary services from survey of 103 dental practices, Ireland, 2020. Source: Author’s own calculations based on desk-based survey of dental practice websites, see “Methods” for full description

For each primary service, the mean is higher than the median suggesting some degree of skewness in the data. Similar to the preventive services, there is more variation in prices across the sample for the more expensive primary services (in particular large composite fillings) as illustrated by the larger size of the boxes and longer whiskers in the boxplots displayed in Fig. 3. The removal of outlying values reduces the mean price for each of these services by between 0.25% (routine extraction) and 5% (large composite filling).

#### Complex services

Table 4 presents the prices of a range of complex services. The mean price for a surgical extraction is €173 (std. €52). The mean price for teeth whitening (take-home kit) is €269 (std. €55). Mean prices for root canal treatment increase by type of tooth from €368 (std. €89) for an incisor tooth, to €463 for a pre-molar tooth (std. €98), to €618 (std. €117) for a molar tooth.^10^

**Table 4:** Adult prices of complex services from survey of 103 dental practices, Ireland, 2020

Complex service	Sample size (*n*)	Price €			
		Min	Max	Median	Mean (std)
Extraction (surgical)	97	80	325	165	173 (52.4)
Teeth whitening	92	150	450	263	269 (55.4)
Endodontics (root canal treatment):					
Incisor	97	150	700	350	368 (88.8)
Pre-molar	95	205	875	450	463 (97.5)
Molar^a^	92	400	975	600	618 (117)

Source: Author’s own calculations based on desk-based survey of dental practice websites, see “Methods” for full description

^a^Where no upper price limit was provided (e.g. ‘from €600’) the price stated was taken as the mid-point price

**Fig. 4:**
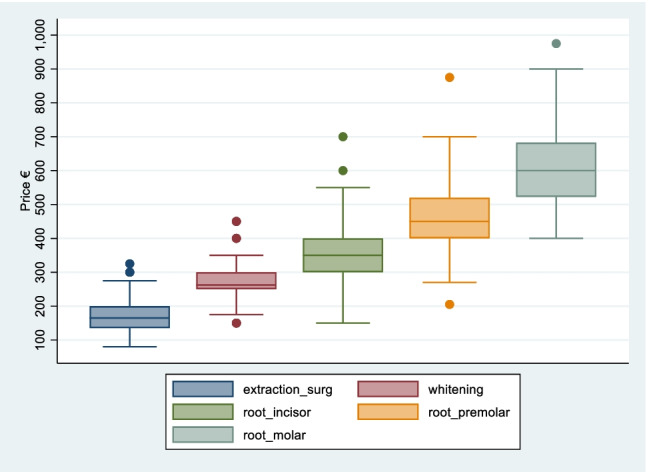
Boxplot of prices for complex services from survey of 103 dental practices, Ireland, 2020. Source: Author’s own calculations based on desk-based survey of dental practice websites, see “Methods” for full description

The mean prices are larger than the median prices indicating skewness in the distribution of prices across the sample. There is more variation in the prices for root canal treatment than for surgical extractions and teeth whitening kits as illustrated in Fig. 4. The removal of outlying values reduces the mean prices for each of these services by between 0.4% (pre-molar root canal treatment) and 2.5% (surgical extraction).

#### Regional analysis

**Fig. 5:**
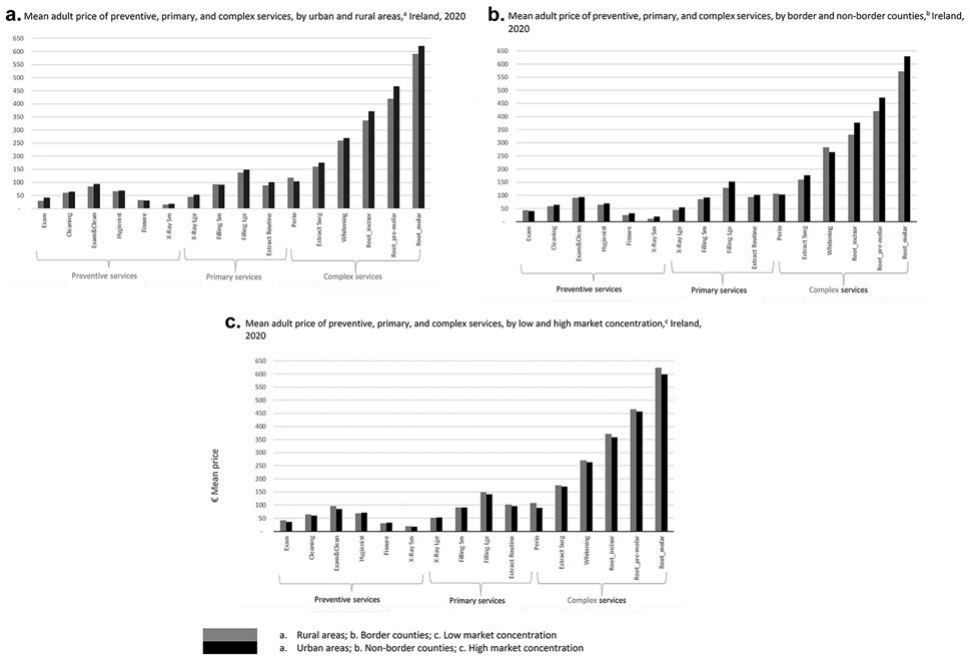
Mean adult price of preventive, primary, and complex services in Ireland: regional patterns. Source: Author’s own calculations based on desk-based survey of dental practice websites, see “Methods” for full description. Notes: ^a^For urban–rural patterns, each dental practice address in the survey was assigned one of six regional categories comprising three urban-based categories and three rural-based categories. For the price comparisons, the six categories were merged into two groups to compare prices between the urban-based and rural-based practices. ^b^For proximity to the border, price comparisons were made between the dental practices located at the border (counties Cavan, Donegal, Leitrim, Louth, Monaghan, and Sligo) and the rest of the country. ^c^For market concentration, the number of dentists per head of population in each county was used as an indicator of market concentration. Low and high market concentrations were defined in relation to the national average number of private dentists per 1000 population (i.e. below and above 0.39 dentists per 1000 population)

Figure 5a presents mean prices for urban and rural-based practices separately. The majority of dental practices included in the survey (91%) fall into one of the three urban categories as per the CSO system of classification (city, satellite urban town, independent urban town) and a small number (9%) are in rural areas (rural areas with high/moderate urban influence, highly rural/remote area). For most services (with the exceptions of small fillings and periodontal care), the mean prices are lower in the rural-based dental practices than in the urban practices. The mean price difference is statistically significant for examinations only (*p* < 0.05). Lower overheads in rural areas compared with urban areas may be contributing to these patterns.

Figure 5b compares mean prices for practices based in border counties with those based in non-border counties. Mean prices are lower in dental practices in border counties compared with practices based in non-border counties for most of the services (examinations, periodontal care, teeth whitening kits are exceptions). These patterns suggest that dental practices may be in competition with practices in nearby areas across the border in Northern Ireland. The mean price difference between border and non-border prices is statistically significant for several services including cleaning, fissure sealants, small and large X-rays, and root canal treatment for incisors and pre-molars (*p* < 0.05).

Figure 5c presents mean prices for practices based in areas with low and high market concentration. As might be expected, the mean prices are higher for many services in dental practices that are based in areas with low market concentration, with some exceptions (hygienist, fissure sealants, large X-rays, and small fillings). However, these mean price differences are statistically significant for examinations and combined examination and cleaning services (< 0.05) only.

#### Prices over time

Table 5 presents the findings from the 2020 survey together with those from previous surveys of dental prices in Ireland. Prices fluctuate somewhat between 2010 and 2020 although the overall trend is for these nominal prices to have increased since 2010 with the notable exception of oral examination. The price increases observed for combined exam and cleaning (22%), and for large extractions (18%) are more than triple the overall trend in consumer prices over the same period (5.5%).^11^ However, methods for sample selection and data collection are not consistent across the different surveys and caution is needed in making comparisons.

**Table 5:** Mean adult prices of selected dental services in Ireland 2010–2020^a^

Service	Mean price €				% Change 2010–2020^ g^
	2010	2014	2018	2020	
Preventive services					
Oral examination	44	45	46	41	−7%
Prophylaxis/cleaning	61	55	64	63	3%
Examination and prophylaxis/cleaning	76			93	22%
Cleaning with hygienist				69	
Fissure sealants (per tooth)				31	
Small X-ray				18	
Large X-ray				52	
Primary services					
Composite fillings (restoration):					
Small		72^b^	81^d^	91	
Large				148	
Extraction (routine)	82	76^c^	85^c^	100	18%
Periodontal (gum) care				104	
Complex services					
Extraction (surgical)				173	
Teeth Whitening		262^e^	281^e^	269	
Endodontics (root canal treatment):					
Incisor			348^f^	368	
Pre-molar				463	
Molar				618	

Sources: For 2010, 2014, 2018: [15–17]

For 2020: Author’s own calculations based on desk-based survey of dental practice websites, see “Methods” for full description

^a^The 2020 survey focuses on prices for adults and this is assumed to be the case for the previous surveys although this is not indicated in the methods for the 2010, 2014, and 2018 surveys

^b^Type of filling not indicated in the source survey, assumed to be small composite for purposes of comparison

^c^Type of extraction not indicated in the source survey, assumed to be routine for purposes of comparison

^d^Size of filling not indicated in the source survey, assumed to be small for purposes of comparison

^e^The 2014 survey does not indicate the type of teeth whitening procedure; the 2018 survey refers to ‘in clinic teeth whitening’. These may not be directly comparable with the 2020 data which refer to the price of a take-home teeth whitening kit

^f^Type of tooth/canal not specified in the source survey

^g^For comparison, Consumer Price Inflation (CPI) over the time period August 2010 to August 2020 was 5.5% (https://www.cso.ie/en/interactivezone/visualisationtools/cpiinflationcalculator/ [last accessed 30/09/2021]

## Discussion

### Core findings

#### Comparisons across the country and services

The results show that both dental supply and dental prices vary across the country and that there exist regional price patterns. Perhaps the most distinctive price differences are between border and non-border areas with prices in the border-based dental practices significantly lower compared with the non-border areas for many of the services. Although beyond the scope of this paper, it would be useful to compare dental prices between Ireland and Northern Ireland and to examine the likely implications for dental care utilisation practices in and around border areas. The results of such a comparison would not be straightforward given the supports offered dentists in the North in the form of capital grants as well as capitation payments. However, the impact on prices for dentists in border areas of physical proximity to Northern competitors does seem obvious and further research could examine this in more detail.

Prices are also typically lower in rural areas compared with urban areas and there is some evidence that prices in areas with high market concentration are lower than in less-concentrated markets. These patterns may be due to price competition but as with the role of the border, however, these results require further research given that other factors, such as higher overhead and rental/building costs in urban areas, may also contribute to price differences.

There are also some differences in the distribution of prices across services wherein the more expensive the treatment, the greater the variation in prices across dental practices. Dentists are reimbursed set amounts for core preventive and primary services under the DTSS and DTBS and this is likely to be influencing the uniformity of prices observed for these services. Conversely, State reimbursement is not available under the DTBS for more expensive treatments such as root canal treatments and less than 6% of claims under the DTSS are for complex treatments that require prior HSE approval.

It is also important to consider that many of these prices refer to specific units of service (e.g. X-rays) and that a course of treatment might involve multiples of these units. For example, a routine dental check-up could include examination, cleaning, and two bitewing X-rays (one for each side of the mouth). The course of recommended treatment can also vary by dentist. Thus, the total price paid by patients depends on both the unit prices (which vary by dental practice) and the combination of services required (which vary from one dentist to another). In addition, the price may vary with volume. For example, some price lists indicate a “package” price for 4 fissure sealants which offers a discount on unit price. This could also be the case for other procedures such as extractions, fillings, and root canal treatments but further details on this is not consistently provided in the price lists included in the survey.

#### Comparisons over time

Assessing the trend in dental prices is complicated by there being insufficient detail on the methods and data sources for previous surveys. The 2014 and 2018 surveys [17, 18] are market-based surveys that do not give specific information on the methods for sample selection or data collected (e.g. type of extraction, type of filling, dentist vs hygienist). There is more clarity and consistency in the approach between the current survey and the 2010 survey by the National Consumer Agency [16] and it can be seen that prices for three core services of cleaning, combined examination and cleaning, and routine extractions have increased by 3–22% while the price of examinations has fallen by 7% over the last 10 years. While these prices changes are out of step with general inflation of 5.5% over the same time period,^12^ health prices in Ireland, including dental prices, are known to be high relative to economy-wide prices and internationally [19].

### Dental pricing patterns

There was notable variation across the dental practices in this sample in terms of the amount of information provided in the price lists. The Dental Council of Ireland specifies a list of services for which prices must be displayed from preventive treatments through to complex services and includes all of the services covered in this survey with the exception of teeth whitening.^13^ While this code of practice refers to the display of a physical price list in the dental surgery (e.g. waiting room, reception area, entrance), it is interesting that is not being fully adhered to in the online price lists. A price was identified for 100% of dentists in the sample for oral examination only. For the remainder of the preventive services, prices were missing from the price lists for up to 51% of the sample in some cases. There were fewer missing observations for primary and complex services with missing observations for between 3 and 38% of the sample. The missing observations may be an indicator that those services are unavailable at those dental practices and this may be the case for some of the more specialist services such as cleaning with a hygienist, or teeth whitening. However, incomplete price lists may explain the missing observations for some of the preventive, primary, and complex services included in this survey. It is also worth noting that this survey initially sets out to assess prices for children/students as well as for adults but the details on prices for these groups were limited to only a handful of price lists.

### Limitations

When sampling the data, not all of the dental practices had an online presence, and thus, there may be some bias in the selection of dental practices. It is also possible that the price data as advertised on websites may not be kept fully up-to-date, and/or the regularity with which price data are updated may vary by dental practice. This is not controlled for in this study but out of a random selection of 10 websites that have been revisited since the survey was completed in August 2020, 70% have been modified in some way (e.g. individual price changes rather than complete revisions of all prices). This suggests in the main, where a dental practice has an online presence, prices are kept up to date and by implication, surveys of such prices need to be conducted on a more frequent basis than has been the case to date.

While the rural–urban indicator was derived from dental practice addresses, the measure of market concentration is a relatively high-level variable based on county-level data on the number of dentists per capita and is itself a crude indicator for competition. A more refined measure was beyond the scope of this paper but could be considered for future analysis.

It is important to point out that in this paper we studied reported prices, not costs, and these should not be confused. For example, there are interesting variations in the prices paid by patient type with the reimbursement price for prophylaxis/cleaning ranging from €31 under the DTSS (in respect of medical cardholders) to €42 under the DTBS (in respect of patients with sufficient PRSI contributions) although dentists can charge up to a maximum of €57 under the scheme, compared with the average price of €63 for private patients observed in this survey. This example highlights the importance in identifying the underlying costs of delivering dental services to make sense of different reimbursement/pricing decisions and identify any potential cross-subsidisation patterns.

There may also be variation across the dental practices in what is covered by each price. Where possible, the prices were adjusted to be consistent across the sample (e.g. a 45-min session with a hygienist was adjusted pro rata to reflect 30-min session), but this was not always possible. For example, anaesthesia services may or may not be charged for separately (e.g. for extractions, periodontal care) and this was not clearly indicated on all lists which makes it more difficult for consumers to make accurate comparisons. Moreover, for some of the more complex services, in particular root canal treatment, the upper limit on prices was not always specified or the price was indicated to vary depending on the complexity of the case, although this phenomenon was more common for procedures such as crowns, dentures, and cosmetic procedures than for the dental services included in this study.

Finally, the data were collected in 2020 and the impact of the COVID-19 pandemic on pricing has not been examined in detail. Anecdotally, dental practices have varied in how charges to cover the additional costs of personal protective equipment (PPE) have been applied with some incorporating these charges into the standard price list, and others applying them as separate charges. Future analysis can assess the extent to which these pricing adjustments persist even after the risks of COVID-19 have abated.

## Conclusions

The new national oral health policy in Ireland acknowledges the importance of oral health for overall well-being and aims to reduce oral health inequalities across the population. However, the main focus of the policy is in improving access for children, adult medical cardholders, and vulnerable patients. Although there is some debate around the level of access envisaged for the rest of the population, this group has even less access to publicly subsidised preventive dental services and pays out-of-pocket for many dental services. This paper provides an up-to-date profile of average private dental prices for core preventive, primary, and complex dental care services, finding that in addition to there being an uneven supply of dentists across the country, dental prices also vary with some notable variations by region and type of service. These factors need to be considered when planning how to improve access to oral healthcare for all individuals in Ireland.

## Footnotes

1 ISAs were regional structures established by the HSE in 2010 with the intention of dividing the health system into 17 ISAs. The ISA structure was never fully completed and has since been replaced by nine Community Healthcare Organisations (CHOs) [10].

2 Medical cards are granted mainly on the basis of an income-based means test (with some non means-tested exceptions) and entitle cardholders to a range of publicly financed healthcare services. Approximately 42% of the population currently hold a medical card or GP Visit card [13]

3 https://www.gov.ie/en/service/1fb655-treatment-benefit-scheme/ [last accessed 03/06/2021].

4 https://www.revenue.ie/en/personal-tax-credits-reliefs-and-exemptions/health-and-age/health-expenses/dental-expenses.aspx [last accessed 04/06/2021].

5 This includes a small number of orthodontists. There is a small discrepancy between the sampling frame (which includes orthodontists) and the sample selected (which excludes orthodontists).

6 Prior to the establishment of the ISAs, the country was divided into 32 Local Health Offices for delivery of community health services.

7 Commercial surveys indicate much larger sample sizes but do not specify the total number, the breakdown by county, or the methods for sample selection [17, 18].

8 In line with national and international policy to phase down the use of amalgam fillings [7], the focus here is on alternative composite (also referred to as white or tooth-coloured) fillings.

9 https://www.cso.ie/en/releasesandpublications/ep/p-urli/urbanandrurallifeinireland2019/introduction/#d.en.211130 [last accessed 02/07/2021].

10 In some price lists, root canal treatment prices were disaggregated by number of canals. We assumed that 1 canal/nerve is comparable with prices for an incisor tooth, 2 canals/nerves with a pre-molar tooth and 3 + canals/nerves with a molar tooth.

11 https://www.cso.ie/en/interactivezone/visualisationtools/cpiinflationcalculator/ [last accessed 30/09/2021].

12 https://www.cso.ie/en/interactivezone/visualisationtools/cpiinflationcalculator/ [last accessed 30/09/2021].

13 http://www.dentalcouncil.ie/displayoffees.php [last accessed 15/07/2021].
